# Effectiveness of Internet-Based or Mobile App Interventions for Family Caregivers of Older Adults with Dementia: A Systematic Review

**DOI:** 10.3390/healthcare12151494

**Published:** 2024-07-27

**Authors:** Fabiane Elizabetha de-Moraes-Ribeiro, Sara Moreno-Cámara, Henrique da-Silva-Domingues, Pedro Ángel Palomino-Moral, Rafael del-Pino-Casado

**Affiliations:** 1 Postgraduate Program in Global Health and Sustainability, University of Sao Paulo, Sao Paulo 05508-220, Brazil; fabianeribeiro04@gmail.com; 2 Department of Nursing, Faculty of Health Sciences, University of Jaén, 23071 Jaén, Spain; smcamara@ujaen.es (S.M.-C.); pamoral@ujaen.es (P.Á.P.-M.); rdelpino@ujaen.es (R.d.-P.-C.)

**Keywords:** aged, internet-based intervention, mobile applications, dementia, caregivers

## Abstract

Introduction: Global aging presents socioeconomic and health challenges. Dementia, a growing concern, affects millions of older adults, intensifying the burden on family caregivers. E-health interventions offer hope through technological solutions, although current research is limited. This study evaluated the effectiveness of internet-based or mobile app interventions for family caregivers of older adults with dementia. Methodology: A systematic review with a narrative synthesis was conducted using databases (PubMed, CINAHL, Scopus, LILACS, and PsycInfo) and the bibliographies of retrieved articles, with no restrictions on time or language. Results: The search yielded 2092 results, of which 22 studies met the inclusion criteria, encompassing a total of 2761 family caregivers. Twenty-one different outcomes were evaluated and classified into three main types of interventions: psychoeducational, psychotherapeutic, and multicomponent. Conclusions: The study highlights the importance of internet-based and mobile app interventions in supporting family caregivers of older adults with dementia. These interventions positively affect many aspects of caregiver well-being, suggesting their utility in addressing this group’s emotional, social, and self-care needs.

## 1. Introduction

Population aging is a global and growing phenomenon that represents a significant achievement for humanity while posing a great challenge. The global population aged 65 and over is projected to increase from 10% in 2022 to 16% in 2050, reaching double the number of children under 5 years old [1]. Addressing the growing needs arising from disabilities and dependency associated with the aging process challenges any society, even those with politically, democratically, and financially stable organizations. Moreover, this situation is intensified when the goal is to achieve healthy aging by maintaining functional and psychological capacity as much as possible throughout life [1,2].

In this demographic and epidemiological scenario of frequent chronic and degenerative diseases, dementia stands out as one of the leading causes of disability and dependency among older adults worldwide [3]. Dementia refers to diseases that affect people’s cognitive and behavioral abilities, significantly interfering with their capacity to carry out daily activities [3]. According to the World Health Organization (WHO) [4,5], more than 55 million people (8.1% of women and 5.4% of men over the age of 65) live with dementia. This number is expected to increase to 78 million by 2030 and 139 million by 2050 [4,5].

Family caregiving refers to unpaid support provided by family members or other individuals close to the dependent. It is the cornerstone of care for older adults in industrialized countries and many other contexts [6,7]. Previous studies concluded that caring for a dependent older adult is associated with high levels of subjective burden, anxiety, and depression, as well as physical ailments [3,4,5,8,9,10,11], which intensify when the person has dementia [12,13,14]. This is possibly due to the high and changing care demands of affected individuals, especially the cognitive-behavioral problems characteristic of this disease [13,14]. 

The scientific literature reveals that conventional interventions to support caregivers of older adults with dementia do not adequately meet their needs [15,16]. However, technology-based interventions can improve care for this group by alleviating stress, reducing workload, optimizing care time, restoring emotional energy, and improving quality of life, among other aspects [17,18].

The WHO [19] defines the use of information and communication technologies (internet or mobile applications) for health purposes as e-health. This reflects the transformation of traditional healthcare models driven by the growing trend of internet usage. Existing interventions have played an important role in supporting families caring for dependent individuals, and this support can be enhanced by leveraging technological innovations.

Despite the promising results highlighted in the scientific literature regarding internet-based or mobile application (e-health) interventions for supporting family caregivers of individuals with dementia, most review studies have significant limitations. For example, one study evaluated the effectiveness of applications available exclusively on specific platforms (Google Play and Apple App Store) [20]. Numerous reviews focused on the effectiveness of internet-based or mobile app interventions by measuring only one or a few outcomes. This is the case with the systematic review conducted by Etxeberria et al. [21], which focused solely on evaluating whether the interventions improved participants’ well-being, based on studies conducted between 2014 and 2018. Similarly, the meta-analysis by Zhao et al. [22] included six studies up to June 2018. It analyzed the impact of these interventions on the mental health of family caregivers of older adults with dementia. Another scoping review [23] focused on three key outcomes: caregiver self-efficacy, depression, and stress/burden. This review included 11 randomized controlled trials (RCTs) up to July 2020 [23]. Furthermore, in the systematic review with meta-analysis (with studies conducted up to January 2020) by Leng et al. [24], the authors found a significant association between internet-based support interventions and only four outcomes (depressive symptoms, perceived distress/stress, anxiety, and self-efficacy).

Therefore, it is crucial at present to implement effective online educational tools and programs aimed at supporting family caregivers of older adults with dementia in their complex role. However, as previously discussed, there is a gap in understanding the effects of internet-based or mobile app interventions. Despite efforts to evaluate these interventions, the lack of confirmed efficacy underscores the need for more comprehensive research. Moreover, there is a requirement for updated evidence of their effectiveness.

Consequently, this study evaluates the effectiveness of internet-based or mobile app interventions for family caregivers of older adults with dementia.

## 2. Materials and Methods

### 2.1. Design

A systematic review with a narrative synthesis was conducted following the recommendations of the Cochrane Handbook of Systematic Reviews [25] and the PRISMA statement [26].

### 2.2. Search Strategy

The search included multiple international electronic databases (PubMed, CINAHL, Scopus, Lilacs, and PsycInfo) using standardized and free terms adapted to each database. Furthermore, a backward citation search of retrieved articles was conducted. Table 1 shows the search strategies used.

### 2.3. Selection of Studies

This review considered the following inclusion criteria: (1) randomized and non-randomized clinical trials; (2) studies conducted with a sample of family caregivers of older adults with dementia; (3) studies involving educational interventions or self-help groups with e-health elements; (4) utilization of a validated measurement instrument; and (5) studies measuring the consequences (positive or negative) of the intervention on family caregivers.

### 2.4. Risk of Bias Assessment 

Potential biases were assessed using an adaptation of the Cochrane Collaboration’s risk of bias assessment tool [27]. This RoB1 tool analyzes study quality by evaluating randomization, allocation concealment, blinding of outcome assessment, and losses to follow-up.

### 2.5. Data Extraction

**Figure 1:**
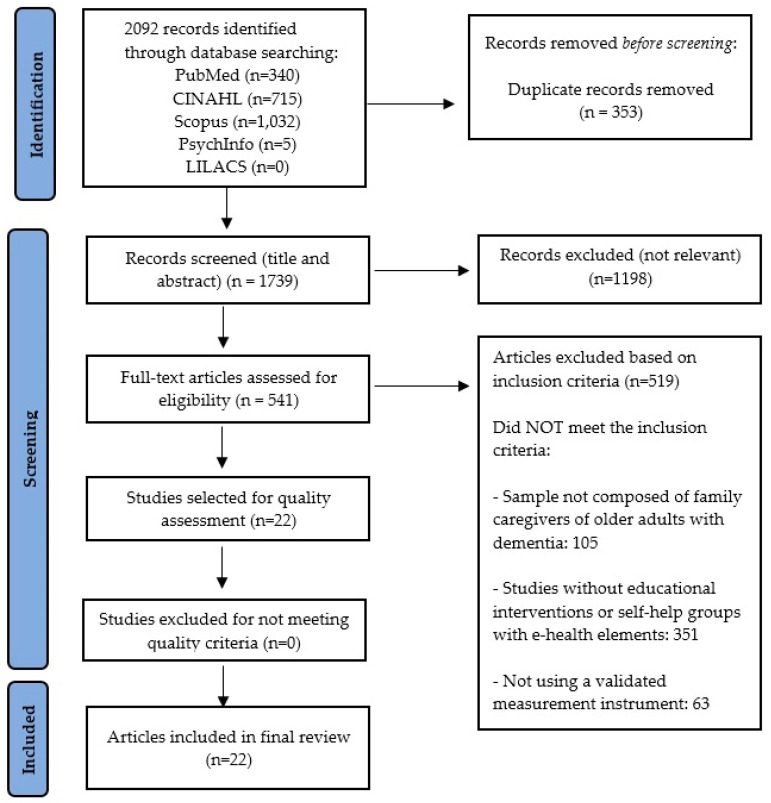
Flowchart. Source: Adapted from Page et al. The PRISMA 2020 statement: an updated guideline for reporting systematic reviews, 2021 [26].

Data were extracted from the studies and collected in a pre-prepared Excel spreadsheet. The collected variables included authors’ names, year and country of publication, study type and sample, e-health element used (internet or mobile applications), instruments used to measure intervention outcomes, and results obtained. Two researchers (F.E.d-M-R. and H.d.-S.-D.) examined the titles and abstracts of all documents to establish their suitability. Any discrepancies between the reviewers were resolved through discussion until a consensus was reached. Additional consultation with the research team was conducted when consensus was not achieved. The reviewers independently reviewed the full texts of selected articles for inclusion, without time or language restrictions. The articles were imported into the EndNote© reference manager for evaluation and selection (Figure 1). 

### 2.6. Data Analysis

The included studies varied in terms of data collection methods and statistical approaches used in analysis. Heterogeneity in measurements and outcomes prevented the application of statistical methods for data synthesis, precluding meta-analysis. Therefore, a narrative synthesis was chosen to gather and summarize information through an iterative process of combining, categorizing, and comparing studies. To facilitate data analysis, measured outcomes were grouped into three categories based on intervention type: (a) psychoeducational therapies [28,29], focusing on teaching skills and promoting autonomy; (b) psychotherapeutic interventions [30,31,32], addressing individual aspects and fostering therapeutic relationships; and (c) multicomponent interventions [30,33,34], combining several approaches. Furthermore, a vote-counting method [26] was employed to contrast the number of studies showing positive versus negative results. Positive votes were assigned to studies demonstrating a statistically significant association between internet-based or mobile app interventions and outcomes related to family caregivers of older adults with dementia. Meanwhile, negative votes indicated no statistical association.

## 3. Results

### 3.1. Search Results

The search strategies yielded 2092 results. After removing duplicate articles and applying the inclusion criteria, 22 studies were included in this systematic review [35,36,37,38,39,40,41,42,43,44,45,46,47,48,49,50,51,52,53,54,55,56]. Figure 1 shows the search and selection process.

### 3.2. Characteristics of Included Studies

Table 2 shows the characteristics of the included studies. All studies were in English. Of these, six were conducted in the United States [35,38,40,43,44,55], four in the Netherlands [37,41,50,53], two in France [39,46], two in the United Kingdom [49,54], one in New Zealand [36], one in Canada [42], one in Germany [45], one in Spain [47], one in South Korea [48], one in Australia [52], one in India [51], and one in Portugal [56]. The total sample of family caregivers across the intervention and control groups was 2317. The studies were published between 1995 and 2023.

#### 3.2.1. Description of Characteristics of Internet-Based or Mobile App Interventions

All interventions included text and/or video functionalities. Over half (12 studies) featured interventions organized into modules [35,36,37,39,41,42,44,45,46,47,51,56]. Other interventions offered multiple functionalities, such as materials and/or activities providing knowledge about the disease [38,40,48,55], information on services supporting caregiving [43,50], and communication spaces and forums [38,43,52,53], among other tools.

Of the 22 studies in this review, 77% reported at least one statistically significant outcome measured in family caregivers. Meanwhile, 23% [39,41,42,45,53] did not report any significant association at the end of the intervention period.

#### 3.2.2. Description of Measured Outcomes in Family Caregivers Following Interventions

**Scheme 1:**
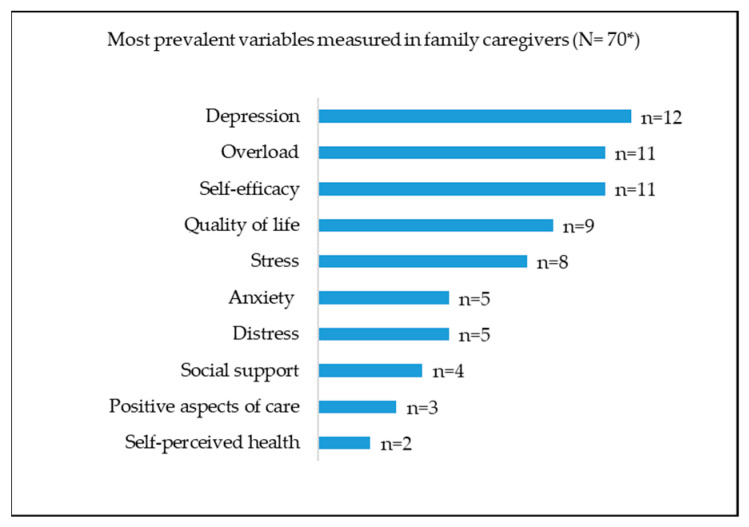
Most assessed outcomes in analyzed interventions. * The total number differs from the number of outcomes because the studies measured more than one outcome.

In total, 21 outcomes were measured across the studies included in our review. The heterogeneity in studies is reflected in the variability in the number of outcomes measured, ranging from those evaluated in a single study to a maximum of 12 studies addressing the same outcome. The most prevalent outcomes were: depression (twelve studies) [35,36,37,39,40,43,44,45,47,51,54,56], caregiver burden (eleven studies) [35,39,40,43,44,46,48,49,51,52,56], self-efficacy (eleven studies) [37,39,41,42,46,47,50,51,53,54,56], quality of life (nine studies) [37,42,44,46,49,50,51,53,56], stress (eight studies) [37,39,44,46,48,53,54,55], anxiety (five studies) [35,36,37,54,56], distress (five studies) [41,49,50,53,54], and social support [38,43,52,53]. Scheme 1 describes the most prevalent outcomes found in the studies. Only one study addressed outcomes such as anticipated grief, confidence in decision-making, coping, decision-making skills, fatigue, hope, perseverance time, person-centered attitude, psychosocial resource utilization, self-care, and sleep efficiency. Thus, they were not included in the graph.

#### 3.2.3. Description of Measurement Instruments Used

Studies used a total of 43 instruments to measure the effectiveness of interventions across various dimensions. The most prevalent were the Center for Epidemiological Studies Depression Scale (CES-D) (nine studies) for assessing depression [35,36,37,40,43,44,45,47,51], the Zarit Burden Interview—ZBI (six studies) for evaluating caregiver burden [39,48,49,51,52,56], and the Hospital Anxiety and Depression Scale—HADS (eight studies) for measuring levels of depression and anxiety [36,37,53,54,56]. Table 2 describes all the instruments found in the studies.

### 3.3. Studies by Type of Interventions

**Scheme 2:**
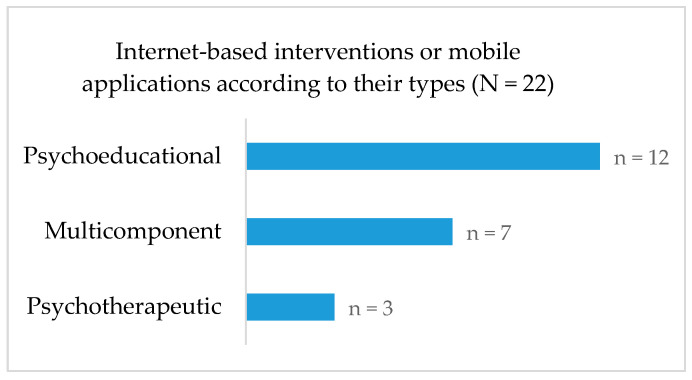
Types of interventions.

The internet-based or mobile application interventions conducted in the studies included in this systematic review were of three types: psychoeducational, which integrates education with psychological therapy, emphasizing the development of skills and the promotion of autonomy [36,37,38,39,41,42,43,44,47,48,51,56]; multicomponent, which uses a combination of several techniques or components to address a problem in a comprehensive manner [35,40,46,49,50,53,55]; and psychotherapeutic, which consists of treating mental health problems and emotional difficulties through dialogue and other psychological techniques, focusing on individual aspects and fostering therapeutic relationships [45,52,54] (Scheme 2). Table 3 shows the systematic review results for each type of outcome and intervention.

#### 3.3.1. Effects of Psychoeducational Interventions

Eight studies [36,37,39,43,44,47,51,56] measured depression. Of these, 37% (three studies) [36,43,47] reported a significant improvement in depressive symptoms (negative association). The five studies with no association had negative outcomes and small sample sizes.

Six studies [39,43,44,48,51,56] assessed caregiver burden. Of these, 33% (two studies) [43,48] reported a reduction in burden (negative association). The four studies with no association had negative outcomes and small sample sizes.

Seven studies [37,39,41,42,47,51,56] measured self-efficacy. Of these, 14% (one study) [37] reported an increase in self-efficacy (positive association). The six studies with no association had positive outcomes, and five of them had small sample sizes.

Five studies [37,42,44,51,56] assessed quality of life. Of these, 20% (one study) [37] identified an improvement in quality of life (positive association). The four studies with no association had positive outcomes, and three of them had small sample sizes.

Four studies [37,39,44,48] measured stress. Of these, 25% (one study) [44] reported stress reduction (negative association). The three studies with no association had negative outcomes and small sample sizes.

Three studies [36,37,56] assessed anxiety. Of these, 67% (two studies) [36,56] reported anxiety reduction (negative association). The study with no association had negative outcomes and a small sample size.

Two studies [38,43] measured social support. Of these, 50% (one study) [43] reported an increase in social support (positive association). The study with no association had positive outcomes and a large sample size.

Two studies [47,56] measured positive aspects of caregiving and showed no effect of the intervention (no association). These two studies had positive outcomes and small sample sizes.

Several outcomes have been evaluated in only one study each: decision-making confidence [38]—benefit (positive association); person-centered attitude [51]—benefit (positive association); fatigue [48]—benefit (negative association); decision-making capacity [38]—no association, with a positive direction and small sample size; distress [41]—no association, with a negative direction and small sample size; hope [42]—no association, with a positive direction and small sample size; self-care [43]—no association, with a positive direction and small sample size; self-perceived health [39]—no association, with a positive direction and small sample size; and sleep efficiency [48]—no association, with a positive direction and small sample size.

#### 3.3.2. Effects of Multicomponent Interventions

Two studies [35,40] measured depression, both of which showed no effect of the intervention (no association). Both studies had negative outcomes, and one had a small sample size.

Four studies [35,40,46,49] assessed caregiver burden. Of these, 75% (three studies) [35,40,49] reported a reduction in caregiver burden (negative association). The study with no association had negative outcomes and a small sample size.

Three studies [46,50,53] measured self-efficacy. Of these, 33% (one study) [50] reported an increase in self-efficacy (positive association). The two studies with no association had positive outcomes and small sample sizes.

Four studies [46,49,50,53] assessed quality of life. Of these, 25% (one study) [49] identified an improvement in quality of life (positive association). The three studies with no association had positive outcomes and small sample sizes.

Three studies [46,53,55] measured stress. Of these, 66% (two studies) [46,53] demonstrated a significant reduction in stress levels (negative association). The study with no association had negative outcomes and a small sample size.

Three studies [49,50,53] measured distress, none of which showed any effect of the intervention (no association). These studies had negative outcomes and small sample sizes.

Several outcomes were evaluated in only one study each: anxiety [35]—benefit (negative association); positive aspects of caregiving [35]—benefit (positive association); coping [35]—no association, with a negative direction and small sample size; perseverance time [53]—no association, with a positive direction and small sample size; and social support [53]—no association, with a positive direction and small sample size.

#### 3.3.3. Effects of Psychotherapeutic Interventions

Two studies [45,54] measured depression. Of these, 50% (one study) [54] showed improvement in depressive symptoms (negative association). The study with no association had negative outcomes and a small sample size. 

Several outcomes were evaluated in only one study each: social support [52]—benefit (positive association); distress [54]—benefit (negative association); stress [54]—benefit (negative association); anticipated grief [45]—no association, with a positive direction and small sample size; anxiety [54]—no association, with a negative direction and large sample size; caregiver burden [52]—no association, with a negative direction and small sample size; psychosocial resource utilization [45]—no association, with a positive direction and small sample size; self-efficacy [54]—no association, with a positive direction and large sample size; and self-perceived health [54]—no association, with a positive direction and large sample size.

### 3.4. Risk of Bias Assessment

**Figure 2:**
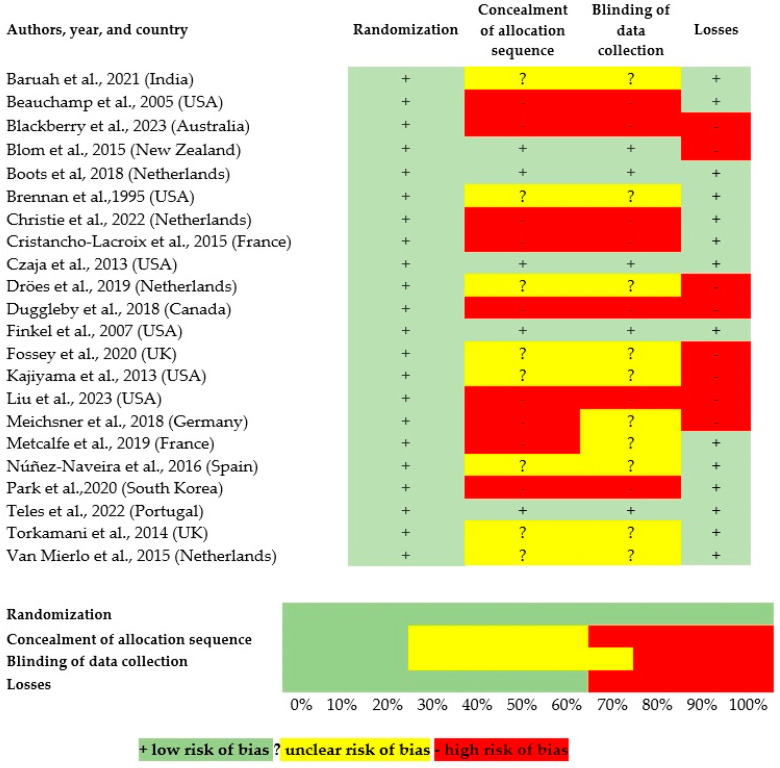
Assessment of risk of bias in included articles. Beauchamp et al., 2005 [35]; Blom et al., 2015 [36]; Boots et al, 2018 [37]; Brennan et al.,1995 [38]; Cristancho-Lacroix et al., 2015 [39]; Czaja et al., 2013 [40]; Dröes et al., 2019 [41]; Duggleby et al., 2018 [42]; Finkel et al., 2007 [43]; Kajiyama et al., 2013 [44]; Meichsner et al., 2018 [45]; Metcalfe et al., 2019 [46]; Núñez-Naveira et al., 2016 [47]; Park et al.,2020 [48]; Torkamani et al., 2014 [49]; Van Mierlo et al., 2015 [50]; Baruah et al., 2021 [51]; Blackberry et al., 2023 [52]; Christie et al., 2022 [53]; Fossey et al., 2020 [54]; Liu et al., 2023 [55]; Teles et al., 2022 [56].

The analysis of biases indicated that 100% of the included studies had a low risk regarding randomization. Regarding concealment of the allocation sequence, 20% of the studies had a low risk, 40% showed an unclear risk, and 40% had a high risk. Regarding blinding of data collection, 20% of the studies demonstrated a low risk, 50% showed an unclear risk, and 30% showed a high risk. Regarding losses in the sample, 60% of the studies were classified as low risk, while 40% were categorized as high risk (Figure 2).

## 4. Discussion

This systematic review provides a comprehensive update on the available evidence regarding the effectiveness of web-based interventions and mobile applications aimed at caregivers of elderly individuals with dementia. We analyzed 22 randomized controlled trials (RCTs) that met the predefined inclusion criteria. Each included study implemented several interventions, classified into three groups based on their characteristics: psychoeducational, psychotherapeutic, and multicomponent. Psychoeducational interventions were the most prevalent [36,37,38,39,41,42,43,44,47,48,51,56]. The studies evaluated 21 outcomes, focusing notably on depression, caregiver burden, self-efficacy, quality of life, stress, anxiety, distress, and social support.

Our findings reflect and expand on previous studies’ evidence regarding the importance of key interventions for family caregivers of elderly individuals with dementia [57,58,59]. Furthermore, the WHO emphasizes the significance of addressing this group in its action plan against dementia [8].

Internet-based or mobile application interventions play a crucial role in supporting family caregiving at home, as evidenced by this systematic review and previous studies [60]. Our results indicate that psychoeducational interventions could be beneficial in assisting family caregivers of elderly individuals with dementia in their caregiving role. This approach focuses on providing knowledge, skills, and practical resources to cope with the challenges of caring for people with dementia [28,29]. Previous studies have highlighted their benefits in reducing caregiver depression [61,62]. Although some outcomes included in our review did not show significant associations (e.g., depression in five out of eight studies, caregiver burden in four out of six studies, self-efficacy in six out of seven studies, quality of life in four out of five studies, stress in three out of four studies, anxiety in one out of three studies, and social support in one out of two studies), the direction of results is promising. The lack of significance is often due to insufficient statistical power caused by small sample sizes (in ten out of twelve studies with psychoeducational interventions).

The results from studies on multicomponent interventions for caregivers are consistent in some aspects, supporting the effectiveness of these interventions in alleviating the emotional and physical burdens associated with caregiving [63]. Although some outcomes did not show significant associations (e.g., depression in two out of two studies, caregiver burden in one out of four studies, self-efficacy in two out of three studies, quality of life in three out of four studies, stress in one out of three studies, and distress in all three studies), the direction of results is promising. The lack of statistical association in these studies could also be attributed to insufficient statistical power due to small sample sizes (in six out of seven studies that employed multicomponent interventions).

Findings from psychotherapeutic interventions have highlighted improvements in depression and perceived social support in certain studies. These results are consistent with previous research demonstrating the efficacy of psychotherapy in reducing depression and promoting social support [64]. Although some outcomes did not show significant associations (e.g., depression in one out of two studies, anticipated grief, anxiety, caregiver burden, and self-efficacy), the direction of results is promising. The lack of statistical association in these studies may also be explained by small sample sizes (in two out of three studies that used psychotherapeutic interventions).

Despite their potential, the studies included in this systematic review show diverse results regarding the effectiveness of internet-based interventions or mobile applications. On the one hand, further research is necessary to understand which components of these interventions are most effective in different caregiver contexts and populations. This analysis underscores the importance of addressing the complexity of caregivers’ experiences and adapting interventions accordingly, recognizing the varied needs and challenges they face [65]. On the other hand, more evidence of statistically significant results has been found with psychoeducational and multicomponent interventions, consistent with previous studies [66,67,68,69]. One possible explanation could be the limited number of studies implementing internet-based psychotherapeutic interventions or mobile applications in this population. Authors such as Andersson and Titov [70] highlight that internet-based psychotherapy is still in a relatively early research stage compared to traditional in-person therapies. They emphasize the need for more studies to fully understand its impact and identify factors contributing to its success or limitations. Furthermore, although internet-based interventions or mobile applications play a significant role, it is essential to recognize that in-person support remains necessary in situations where it is required.

We observed variability in the risk of bias among studies included in our research. While randomization proved robust in all cases, with 100% of studies classified as low risk, variations were identified in other domains. Concealment of allocation sequence and blinding of outcome assessment showed diversity in risk, with a percentage of studies classified as unclear or high risk. Moreover, the importance of properly handling sample losses was emphasized, with a percentage of studies identified as high risk in this domain. These findings underscore the need for greater attention to transparency and methodological rigor in future research to ensure the validity and reliability of overall results.

Regarding limitations, excessive heterogeneity among interventions, outcomes, and assessment instruments used to evaluate the effects of internet-based or mobile application interventions on family caregivers of elderly individuals with dementia complicates comparisons between interventions and hinders more robust and precise analyses, such as meta-analyses. Furthermore, the small sample sizes in most studies, the lack of statistical association in some outcomes, the absence of psychotherapeutic interventions, and the complex nature of family caregiving pose challenges for a more comprehensive interpretation. These factors underscore the need for future personalized interventions tailored to individual caregiver needs. Nevertheless, a thorough peer-reviewed analysis of several interventions has been conducted to classify them, along with the results and measurement instruments used.

Therefore, for future research, further research is needed on different interventions based on internet-based or mobile application interventions, especially considering variables such as cultural context, level of experience, and individual caregiver characteristics, to reach more definitive conclusions that can guide strategies to support and care for family caregivers in their roles.

## 5. Conclusions

Our systematic review provides comprehensive and detailed insight into internet-based and mobile application interventions aimed at caregivers of elderly individuals with dementia. The analysis identified 22 studies, categorizing them into three predominant intervention types: psychoeducational, multicomponent, and psychotherapeutic. We identified 21 outcomes evaluated across the examined studies, with the most prevalent being depression, caregiver burden, self-efficacy, quality of life, stress, anxiety, distress, and social support. This diversity reflects the inherent complexity of caring for elderly individuals with dementia and the multitude of dimensions that can influence the experience of family caregivers.

While some studies found no statistically significant associations between certain outcomes, the direction of these results was promising. This suggests that internet-based interventions or mobile applications may have potential benefits. However, in many cases, small sample sizes may have limited the ability to demonstrate statistically significant associations.

Our findings underscore the ongoing importance of research and the development of tailored and effective interventions that can provide meaningful support to family caregivers in their daily caregiving responsibilities.

## Figures and Tables

**Table 1:** Databases, search strategies, and records.

Database	Search String	Records Retrieved December 2023
Pubmed	(Caregivers[mh] OR ((Caregiver*[tiab] OR Carer*[tiab] OR Caregiv*[tiab] OR Care giv*[tiab]) not medline[sb])) AND ((Dementia[mh:noexp] OR Alzheimer Disease[mh]) OR ((Dement*[tiab] OR Alzheimer*[tiab] OR Alzheimer’s disease[tiab]) not medline[sb])) AND ((Mobile applications[mh] OR Telemedicine[mh] OR Social Media[mh] OR Social Networking[mh] OR Social Support[mh] OR Internet[mh] OR Web browser[mh]) OR ((Mobile app*[tiab] OR Portable Electronic App*[tiab] OR App[tiab] OR Portable Software App*[tiab] OR Portable Software*[tiab] OR telemedicine[tiab] OR mobile health[tiab] OR mHealth[tiab] OR telehealth[tiab] OR eHealth[tiab] OR Mobile technology[tiab] OR Social medi*[tiab] OR Social Network*[tiab] OR Social suppor*[tiab] OR Online Social support*[tiab] OR online* OR Internet[tiab] OR Web browser*[tiab]) not medline[sb])) AND ((Treatment Outcome[mh] OR Efficiency[mh]) OR ((Treatment Outcome*[Tiab] OR Efficiency[Tiab] OR Intervention[Tiab] OR Intervent*[Tiab] OR Treatment effectiveness[Tiab] OR effectiveness[Tiab] OR Treatment efficacy[Tiab] OR efficacy[tiab]) not medline[sb]))	340
CINAHL	(MH Caregivers OR AB Caregiver* OR AB Carer* OR AB Caregiv* OR AB Care giv*) AND (MH Dementia OR MH Alzheimer Disease OR AB Dement* OR AB Alzheimer* OR AB Alzheimer’s disease) AND (MH Mobile applications OR MH Telemedicine OR MH Social Media OR MH Social Networking OR MH Social Support OR MH Internet OR MH Web browser OR AB Mobile app* OR AB Portable Electronic App* OR AB App OR AB Portable Software App* OR AB Portable Software* OR AB telemedicine OR AB mobile health OR AB mHealth OR AB telehealth OR AB eHealth OR AB Mobile technology OR AB Social medi* OR AB Social Network* OR AB Social suppor* OR AB Online Social support* OR AB online* OR AB Internet OR AB Web browser*) AND (MH Treatment Outcome OR MH Efficiency OR AB Treatment Outcome* OR AB Efficiency OR AB Intervention OR AB Intervent* OR AB Treatment effectiveness OR AB effectiveness OR AB Treatment efficacy OR AB efficacy)	715
Scopus	(KEY(caregivers) OR TITLE-ABS (caregiver*) AND KEY (dementia OR “Alzheimer Disease”) OR TITLE-ABS (dement* OR alzheimer*) AND KEY (“Mobile applications” OR telemedicine OR “Social Media” OR “Social Networking” OR “Social Support” OR internet OR “Web browser”) OR TITLE-ABS (mobile AND app* OR portable AND electronic AND app* OR app OR portable AND software AND app* OR portable AND software* OR telemedicine OR “mobile health” OR mhealth OR telehealth OR ehealth OR “Mobile technology” OR social AND medi* OR social AND network* OR social AND suppor* OR online AND social AND support* OR online* OR internet OR web AND browser*) AND KEY (“Treatment Outcome” OR efficiency) OR TITLE-ABS (“Treatment Outcome*” OR efficiency OR intervention OR intervent* OR “Treatment effectiveness” OR effectiveness OR “Treatment efficacy” OR efficacy))	1.032
LILACS	(TW:(Cuidadores OR Cuidadores Familiares OR Cuidador$ Familia$ OR Familia$ Cuidador$)) AND TW:(Demencia OR Enfermedad de Alzheimer OR Demencia Tipo Alzheimer OR Demencia de Alzheimer OR Mal de Alzheimer) AND TW:(Aplicaciones Móviles OR Tecnología OR Tecnología y Aplicaciones de Software OR Telemedicina OR Salud Digital OR Salud Eletrónica OR Salud Mueble OR eSalud OR Servicios de Telesalud OR Servicios de eSalud OR Servicios en Telemedicina OR Telesalud OR Ciber Salud OR Internet OR Medios de Comunicación Sociales OR Programas Informaticos OR Aplicacion$ de Softwar$ OR Herramientas de Software) AND (TW:(Eficacia OR Eficiencia OR Resultado del Tratamiento OR Eficacia del Tratamiento))	0
PsycINFO	(MJSUB(Caregivers) OR AB(elder care)) AND (MJSUB(Dementia) OR MJSUB(Alzheimer’s Disease)) AND (MJSUB(Mobile Applications) OR MJSUB(Telemedicine) OR MJSUB(Digital Technology) OR MJSUB(Mobile Health) OR MJSUB(Electronic Health Services) OR MJSUB(Internet) OR MJSUB(Computer Applications) OR MJSUB(Electronic Health Services) OR MJSUB(Digital Interventions)) AND (MJSUB(Treatment Effectiveness Evaluation) OR MJSUB(Treatment Outcomes) OR MJSUB(Employee Efficiency))	5

**Table 2:** Characteristics of Included Studies.

Authors, Year, and Country	Type of Study	Sample	Type of Intervention	Instruments	Results After Intervention in the Intervention Group
Baruah et al., 2021 (India) [51]	Design: RCT Duration: 3 months	Intervention: 29 Control: 26	Psychoeducational	ADQ; CES-D10; ZBI; RIS; EuroQol-VAS.	Increased person-centered attitude toward the person with dementia (*p* = 0.030). No significant differences were observed in depression (*p* = 0.630), burden (*p* = 0.119), self-efficacy in elder carely (*p* = 0.779), and quality of life (*p* = 0.214).
Beauchamp et al., 2005 (USA) [35]	Design: RCT Duration: 30 days	Intervention: 150 Control: 149	Multicomponent	CSI; PACS; STAI;Revised Ways of Coping; CES-D.	Reduction in burden (*p* = 0.028). Increase in positive aspects of caregiving (*p* = 0.021). Anxiety reduction (*p* = 0.030). No significant differences were observed in coping (*p* = 0.971) or depression symptoms (*p* = 0.090).
Blackberry et al., 2023 (Australia) [52]	Design: RCT Duration: 32 weeks	Intervention: 37 Control: 32	Psychotherapeutic	MOS-SSS; ZBI.	Significant improvement in social support (*p* = 0.003). No significant differences were observed in burden (*p* = 0.090).
Blom et al., 2015 (New Zealand) [36]	Design: RCT Duration: 6 months	Intervention: 149 Control: 96	Psychoeducational	HADS-A; CES-D.	Reduction in anxiety symptoms (*p* = 0.007) and depression symptoms (*p* = 0.034).
Boots et al., 2018 (Netherlands) [37]	Design: RCT Duration: 8 weeks	Intervention: 41 Control: 40	Psychoeducational	CSES; ICECAP-O; PSS; HADS-A; CES-D.	Increased caregiver self-efficacy (care management and service use) (*p* = 0.002) and quality of life (*p* = 0.032). No significant differences were observed in stress (*p* = 0.071), anxiety (*p* = 0.374), or depression (*p* = 0.293).
Brennan et al., 1995 (USA) [38]	Design: RCT Duration: 1 year	Intervention: 51 Control: 51	Psychoeducational	Decision confidence scale;Decision-making skills; IESS.	Improvement in decision-making confidence (*p* < 0.01). No significant differences were observed in decision-making skills (*p* = 0.200) or social support (*p* = 0.510).
Christie et al., 2022 (Netherlands) [53]	Design: RCT Duration: 16 weeks	Intervention: 40 Control: 45	Multicomponent	SSCQ; MSPSS; HADS; ICECAP-O; Pt; PSS.	No significant differences were observed in any outcome: self-efficacy (sense of competence) (*p* = 0.870), social support (*p* = 0.620), distress (*p* = 0.310), quality of life (*p* = 0.930), perseverance time (*p* = 0.950), and stress (*p* = 0.670).
Cristancho-Lacroix et al., 2015 (France) [39]	Design: RCT Duration: 3 months	Intervention: 25 Control: 24	Psychoeducational	PSS-14; RSCSE; ZBI; BDI-II; NHP.	No significant differences were observed in any outcome: stress (*p* = 0.980), self-efficacy (obtaining respite, responding to disruptive behaviors, and controlling upsetting thoughts about caregiving) (*p* > 0.05), burden (*p* = 0.740), depression (*p* = 0.560), and perceived health (*p* > 0.05).
Czaja et al., 2013 (USA) [40]	RCT Duration: 5 months	Intervention: 38 Control 1: 36	Multicomponent	RMBPC; CES-D.	Decrease in caregiver burden (*p* = 0.029). No significant difference in depression (*p* = 0.924).
Dröes et al., 2019 (Netherlands) [41]	Design: RCT Duration: 6 months	Intervention: 65 Control: 54	Psychoeducational	NPI; SSCQ.	No significant differences were observed in any outcome: distress (*p* = 0.370) or self-efficacy (sense of competence) (*p* = 0.200).
Duggleby et al., 2018 (Canada) [42]	Diseño: ECADuración: 2 years	Intervention: 101 Control: 98	Psychoeducational	SF-12v2; GSES; HHI.	No significant differences were observed in any outcome: quality of life (*p* = 0.880), self-efficacy (general sense of perceived self-efficacy) (*p* = 0.670), or hope (*p* = 0.170).
Finkel et al., 2007 (USA) [43]	RCT Duration: 6 months	Intervention: 23 Control: 23	Psychoeducational	CES-D; RMBPC; Inventory of Socially Supportive Behaviors; Caregiver health and Health Behaviors Scale.	Improvement in depression (*p* = 0.009). Reduction in caregiver burden (*p* = 0.025), improvement in social support (*p* = 0.043). No significant differences were observed in self-care (*p* = 0.074).
Fossey et al., 2020 (United Kingdom) [54]	Design: RCT Duration: 26 weeks	Intervention: 213 Control: 212	Psychotherapeutic	HADS; HADS-D; RSS; GHQ-12; HADS-A; SSCQ.	Improvement in caregiver distress (*p* = 0.028) and depression (*p* = 0.004), and reduced stress (*p* = 0.040). No significant differences were observed in perceived health (*p* = 0.430), anxiety (*p* = 0.220), or self-efficacy (sense of competence) (*p* = 0.240).
Kajiyama et al., 2013 (USA) [44]	RCT Duration: 3 months	Intervention: 75 Control: 75	Psychoeducational	PSS; CES-D; PQoL; RMBPC.	Decrease in stress (*p* = 0.017). No significant differences were observed in depression (*p* = 0.098), quality of life (*p* > 0.05), or caregiver burden (*p* = 0.600).
Liu et al., 2023 (USA) [55]	Design: RCT Duration: 4 weeks	Intervention: 6 Control: 25	Multicomponent	PSS.	Significantly lower levels of stress were observed (*p* < 0.001).
Meichsner et al., 2018 (Germany) [45]	Design: RCT Duration: 8 weeks	Intervention: 19 Control: 18	Psychotherapeutic	CES-D; CGS; Psychosocial Resource Utilization Questionnaire for Family Caregivers of People with Dementia.	There was no statistical significance in any outcome: depression (*p* = 0.910), anticipatory grief (*p* = 0.125), or utilization of psychosocial resources (*p* > 0.05).
Metcalfe et al., 2019 (France) [46]	Design: RCT Duration: 12 weeks	Intervention: 30 Control: 31	Multicomponent	PSS; RSCSE; RMBPC;EQ-5D-5L.	Reduction in stress levels (*p* = 0.030). No significant differences were observed in self-efficacy (obtaining respite, responding to disruptive behaviors, and controlling upsetting thoughts about caregiving) (*p* > 0.05), caregiver burden (*p* = 0.110), or quality of life (*p* = 0.280).
Núñez-Naveira et al., 2016 (Spain) [47]	Design: RCT Duration: 3 months	Intervention: 30 Control: 31	Psychoeducational	CES-D; CCS; RCSS.	Decrease in depressive symptoms (*p* = 0.037). No significant differences were observed in self-efficacy (sense of competence) (*p* = 0.457) or positive aspects of caregiving (*p* = 0.916).
Park et al., 2020 (South Korea) [48]	Design: Non-randomized controlled trial	Duration: 4 weeks	Psychoeducational	RPFS; ZBI; Saliva cortisol levels; Sleep efficiency.	Reduction in fatigue (*p* = 0.003) and improvement in caregiver burden (*p* < 0.001). No significant differences were observed in stress (*p* > 0.05) or sleep (*p* > 0.05).
Teles et al., 2022 (Portugal) [56]	Design: RCT Duration: 6 months	Intervention: 11 Control: 20	Psychoeducational	HADS-A; HADS-D; ZBI; WHOQOL-BREF; PACS; GSE.	No significant differences were observed in depression symptoms (*p* = 0.347), caregiver burden (*p* = 0.800), quality of life (*p* = 0.973), positive aspects of caregiving (*p* = 0.125), or self-efficacy (competence in caregiving) (*p* = 0.109).
Torkamani et al., 2014 (United Kingdom) [49]	Design: RCT Duration: 6 months	Intervention: 30 Control: 30	Multicomponent	ZBI; EuroQOL; NPI.	Improvement in caregiver burden (*p* = 0.044) and quality of life (*p* = 0.026). No significant differences were observed in caregiver distress (*p* = 0.061).
Van Mierlo et al., 2015 (Netherlands) [50]	Design: RCT Duration: 12 months	Intervention: 41 Control: 32	Multicomponent	SSCQ; EQ5D+c; NPI.	Improvement in self-efficacy (sense of competence) (*p* = 0.010). No statistical significance in quality of life (*p* > 0.05) or distress (*p* > 0.05).

RCT: Randomized Controlled Trial; ADQ: Approaches to Dementia Questionnaire; BDI-II: Beck Depression Inventory; CCS: Caregiver Competence Scale; CES-D: Center for Epidemiological Studies Depression scale; CES-D10: Center for Epidemiological Studies Depression-10 item scale; CGS: Caregiver Grief Scale; CSES: Caregiver Self-Efficacy Scale; CSI: Caregiver Strain Instrument; EQ-5D-5L: Caregiver’s Health-related quality of life; EQ5D-VAS: EuroQol—Euro Quality of life—Visual Analog Scale; EQ5D+c: European Quality of Life-5 Dimensions; EuroQol: Euro Quality of life; GHQ-12: General Health Questionnaire-12; GSE: Generalized Self-Efficacy scale; GSES: General Self-Efficacy Scale; HADS: Hospital Anxiety and Depression Scale; HADS-A: Hospital Anxiety and Depression Scale; HADS-D: Hospital Anxiety and Depression Scale; HHI: Herth Hope Index; ICECAP-O: Investigating Choice Experiments for the preferences of older people CAPability measure for Older people; IESS: Instrumental and Expressive Social Support scale; MOS-SSS: Medical Outcomes Study Social Support Survey; MSPSS: Multidimensional Scale of Perceived Support; NHP: Nottingham Health Profile; NPI: Neuropsychiatric Inventory; PACS: Positive Aspects of Caregiving Survey; PQOL: Perceived Quality of Life scale; PSS: caregiver Perceived Stress Scale; PSS-14: Perceived Stress Scale; Pt: Tiempo de perseverancia; RCSS: Revised Caregiving Satisfaction Scale; RIS: eldercare self-efficacy scale; RMBPC: Revised Memory and Behavior Checklist; RPFS: Revised Piper Fatigue Scale; RSCSE: Revised Scale for Caregiving Self-Efficacy; RSS: Relative Stress Scale; SF-12v2: Short Form-12 item [version 2] health survey; SSCQ: Short Sense of Competence Questionnaire; STAI: State-Trait Anxiety Inventory; WHOQOL-BREF: World Health Organization Quality Of Life Brief version; ZBI: Zarit Burden Interview.

**Table 3:** Characteristics of systematic review results.

Dependent Variable	Independent Variable	k	Outcome			
			Benefit (k)	Harm (k)	No Association (k)	Direction in Studies with No Association
Anticipated grief	Psychotherapy	1	0	0	1	Positive
Anxiety	Psychoeducational	3	2	0	1	Negative
	Multicomponent	1	1	0	0	
	Psychotherapy	1	0	0	1	Negative
Confidence in decision-making	Psychoeducational	1	1	0	0	
Coping	Multicomponent	1	0	0	1	Positive
Decision-making skills	Psychoeducational	1	0	0	1	Positive
Depression	Psychoeducational	8	3	0	5	Negative
	Psychotherapy	2	1	0	1	Negative
	Multicomponent	2	0	0	2	Negative
Distress	Multicomponent	3	0	0	3	Negative
	Psychotherapy	1	1	0	0	
	Psychoeducational	1	0	0	1	Negative
Fatigue	Psychoeducational	1	1	0	0	
Hope	Psychoeducational	1	0	0	1	Positive
Overload	Psychoeducational	6	2	0	4	Negative
	Multicomponent	4	3	0	1	Negative
	Psychotherapy	1	0	0	1	Negative
Perseverance time	Multicomponent	1	0	0	1	Positive
Person-centered attitude	Psychoeducational	1	1	0	0	
Positive aspects of care	Psychoeducational	2	0	0	2	Positive
	Multicomponent	1	1	0	0	
Psychosocial resource utilization	Psychotherapy	1	0	0	1	Positive
Quality of life	Psychoeducational	5	1	0	4	Positive
	Multicomponent	4	1	0	3	Positive
Self-care	Psychoeducational	1	0	0	1	Positive
Self-efficacy	Psychoeducational	7	1	0	6	Positive
	Multicomponent	3	1	0	2	Positive
	Psychotherapy	1	0	0	1	Positive
Self-perceived health	Psychoeducational	1	0	0	1	Positive
	Psychotherapy	1	0	0	1	Positive
Sleep efficiency	Psychoeducational	1	0	0	1	Positive
Social support	Psychoeducational	2	1	0	1	Negative
	Psychotherapy	1	1	0	0	
	Multicomponent	1	0	0	1	Positive
Stress	Psychoeducational	4	1	0	3	Negative
	Multicomponent	3	2	0	1	Negative
	Psychotherapy	1	1	0	0	

k = number of studies.

## Data Availability

Raw data supporting the conclusions of this article will be made available by the authors on request.

## References

[1] United Nations World Population Prospects 2022: Summary of Results 2022. https://www.un.org/development/desa/pd/sites/www.un.org.development.desa.pd/files/undesa_pd_2022_wpp_key-messages.pdf.

[2] United Nations (2019). Department of Economic and Social Affairs. Population Division. World Population Ageing 2019. https://www.un.org/en/development/desa/population/publications/pdf/ageing/WorldPopulationAgeing2019-Highlights.pdf.

[3] World Health Organization (2021). Global Status Report on the Public Health Response to Dementia.

[4] World Health Organization (2017). Global Action Plan on the Public Health Response to Dementia 2017–2025.

[5] World Health Organization (2017). Dementia.

[6] Fujisawa R., Colombo F. (2009). The Long-Term Care Workforce: Overview and Strategies to Adapt Supply to a Growing Demand.

[7] Schulz R., Beach S.R., Czaja S.J., Martire L.M., Monin J.K. (2020). Family Caregiving for Older Adults. Annu. Rev. Psychol..

[8] World Health Organization (2018). Towards a Dementia Plan: A WHO Guide.

[9] Pinquart M.S. (2003). Associations of stressors and uplifts of caregiving with caregiver burden and depressive mood: A meta-analysis. J. Gerontology. Ser. B Psychol. Sci. Soc. Sci..

[10] Kim H., Chang M., Rose K., Kim S. (2021). Predictors of caregiver burden in caregivers of individuals with dementia. J. Adv. Nurs..

[11] Schoenmakers B., Buntinx F., Delepeleire J. (2010). Factors determining the impact of care-giving on caregivers of elderly patients with dementia. A systematic literature review. Maturitas.

[12] Pinquart M., Sörensen S. (2003). Differences between caregivers and noncaregivers in psychological health and physical health: A meta-analysis. Psychol. Aging.

[13] Etters L., Goodall D., Harrison B.E. (2008). Caregiver burden among dementia patient caregivers: A review of the literature. J. Am. Assoc. Nurse Pract..

[14] Fonareva I., Oken B.S. (2014). Physiological and functional consequences of caregiving for relatives with dementia. Int. Psychogeriatr..

[15] Thompson G.N., Roger K. (2014). Understanding the needs of family caregivers of older adults dying with dementia. Palliat. Support. Care.

[16] Stirling C., Andrews S., Croft T., Vickers J., Turner P., Robinson A. (2010). Measuring dementia carers’ unmet need for services--an exploratory mixed method study. BMC Health Serv. Res..

[17] Power J., Wherton J.P., Prendergast D., Lawlor B. (2012). Identifying opportunities for supporting caregivers of persons with dementia through information and communication technology. Gerontechnology.

[18] Adler R., Mehta R. (2014). Catalyzing Technology to Support Family Caregiving.

[19] World Health Organization (2012). National eHealth Strategy Toolkit.

[20] Castillo L.I., Tran V., Hadjistavropoulos T. (2024). Are mobile apps meeting the needs of caregivers of people living with dementia? An evaluation of existing apps for caregivers. Aging Ment. Health.

[21] Etxeberria I., Salaberria K., Gorostiaga A. (2021). Online support for family caregivers of people with dementia: A systematic review and meta-analysis of RCTs and quasi-experimental studies. Aging Ment. Health.

[22] Zhao Y., Feng H., Hu M., Hu H., Li H., Ning H., Chen H., Liao L., Peng L. (2019). Web-based interventions to improve mental health in home caregivers of people with dementia: Meta-analysis. J. Med. Internet Res..

[23] Sztramko R., Levinson A.J., Wurster A.E., Jezrawi R., Sivapathasundaram B., Papaioannou A., Cowan D., St Onge J., Marr S., Patterson C. (2021). Online educational tools for caregivers of people with dementia: A scoping literature review. Can. Geriatr. J..

[24] Leng M., Zhao Y., Xiao H., Li C., Wang Z. (2020). Internet-based supportive interventions for family caregivers of people with dementia: Systematic review and meta-analysis. J. Med. Internet Res..

[25] Centro Cochrane Iberoamericano (2012). Traductores. Manual Cochrane de Revisiones Sistemáticas de Intervenciones.

[26] Page M.J., McKenzie J.E., Bossuyt P.M., Boutron I., Hoffmann T.C., Mulrow C.D., Shamseer L., Tetzlaff J.M., Akl E.A., Brennan S.E. (2021). The PRISMA 2020 statement: An updated guideline for reporting systematic reviews. Int. J. Surg..

[27] Higgins J.P., Altman D.G., Gøtzsche P.C., Jüni P., Moher D., Oxman A.D., Savović J., Schulz K.F., Weeks L., Sterne J.A. (2011). The Cochrane Collaboration’s tool for assessing risk of bias in randomised trials. BMJ.

[28] Authier J. (2012). The Psychoeducation Model: Definition, Contemporary Roots and Content. Can. J. Couns. Psychother..

[29] Lemes C.B., Ondere Neto J. (2017). Aplicações da psicoeducação no contexto da saúde. Temas Em Psicol..

[30] (2007). Instituto de Mayores y Servicios SocialesModelo de Atención a las Personas con Enfermedades de Alzheimer.

[31] National Institute for Health and Care Excellence (2018). Dementia: Assessment, Management and Support for People Living with Dementia and Their Carers.

[32] Sörensen S., Pinquart M., Duberstein P. (2002). How Effective Are Interventions with Caregivers? An Updated Meta-Analysis. Gerontologist.

[33] Fazio S., Pace D., Maslow K., Zimmerman S., Kallmyer B. (2018). Alzheimer’s Association dementia care practice recommendations. Gerontologist.

[34] de Alzheimer C.E. (2018). Terapias no Farmacológicas en las Asociaciones de Familiares de Personas con Alzheimer.

[35] Beauchamp N., Irvine A.B., Seeley J., Johnson B. (2005). Worksite-based internet multimedia program for family caregivers of persons with dementia. Gerontologist.

[36] Blom M.M., Zarit S.H., Groot Zwaaftink R.B.M., Cuijpers P., Pot A.M. (2015). Effectiveness of an internet intervention for family caregivers of people with dementia: Results of a randomized controlled trial. PLoS ONE.

[37] Boots L.M.M., De Vugt M.E., Kempen G.I.J.M., Verhey F.R.J. (2018). Effectiveness of a blended care self-management program for caregivers of people with early-stage dementia (partner in balance): Randomized controlled trial. J. Med. Internet Res..

[38] Brennan P.F., Moore S.M., Smyth K.A. (1995). The effects of a special computer network on caregivers of persons with alzheimer’s disease. Nurs. Res..

[39] Cristancho-Lacroix V., Wrobel J., Cantegreil-Kallen I., Dub T., Rouquette A., Rigaud A.-S. (2015). A web-based psychoeducational program for informal caregivers of patients with Alzheimer’s disease: A pilot randomized controlled trial. J. Med. Internet Res..

[40] Czaja S.J., Loewenstein D., Schulz R., Nair S.N., Perdomo D. (2013). A videophone psychosocial intervention for dementia caregivers. Am. J. Geriatr. Psychiatry.

[41] Dröes R.M., van Rijn A., Rus E., Dacier S., Meiland F. (2019). Utilization, effect, and benefit of the individualized meeting centers support program for people with dementia and caregivers. Clin. Interv. Aging.

[42] Duggleby W., Ploeg J., McAiney C., Peacock S., Fisher K., Ghosh S., Markle-Reid M., Swindle J., Williams A., Triscott J.A. (2018). Web-Based Intervention for Family Carers of Persons with Dementia and Multiple Chronic Conditions (My Tools 4 Care): Pragmatic Randomized Controlled Trial. J. Med. Internet Res..

[43] Finkel S., Czaja S.J., Martinovich Z., Harris C., Pezzuto D., Schulz R. (2007). E-Care: A Telecommunications Technology Intervention for Family Caregivers of Dementia Patients. Am. J. Geriatr. Psychiatry.

[44] Kajiyama B., Thompson L.W., Eto-Iwase T., Yamashita M., Di Mario J., Marian Tzuang Y., Gallagher-Thompson D. (2013). Exploring the effectiveness of an internet-based program for reducing caregiver distress using the iCare Stress Management e-Training Program. Aging Ment. Health.

[45] Meichsner F., Theurer C., Wilz G. (2019). Acceptance and treatment effects of an internet-delivered cognitive-behavioral intervention for family caregivers of people with dementia: A randomized-controlled trial. J. Clin. Psychol..

[46] Metcalfe A., Jones B., Mayer J., Gage H., Oyebode J., Boucault S., Aloui S., Schwertel U., Böhm M., Tezenas du Montcel S. (2019). Online information and support for carers of people with young-onset dementia: A multi-site randomised controlled pilot study. Int. J. Geriatr. Psychiatry.

[47] Núñez-Naveira L., Alonso-Búa B., De Labra C., Gregersen R., Maibom K., Mojs E., Krawczyk-Wasielewska A., Millán-Calenti J.C. (2016). UnderstAID, an ICT Platform to Help Informal Caregivers of People with Dementia: A Pilot Randomized Controlled Study. BioMed Res. Int..

[48] Park E., Park H., Kim E.K. (2020). The effect of a comprehensive mobile application program (CMAP) for family caregivers of home-dwelling patients with dementia: A preliminary research. Jpn. J. Nurs. Sci..

[49] Torkamani M., McDonald L., Aguayo I.S., Kanios C., Katsanou M.-N., Madeley L., Limousin P.D., Lees A.J., Haritou M., Jahanshahi M. (2014). A randomized controlled pilot study to evaluate a technology platform for the assisted living of people with dementia and their carers. J. Alzheimer’s Dis..

[50] Van Mierlo L.D., Meiland F.J.M., Van de Ven P.M., Van Hout H.P.J., Dröes R.-M. (2015). Evaluation of DEM-DISC, customized e-advice on health and social su.support program for caregivers of people with dementia in India: A randomized controlled trial. Int. Psychogeriatr..

[51] Baruah U., Varghese M., Loganathan S., Mehta K.M., Gallagher-Thompson D., Zandi D., Dua T., Pot A.M. (2021). Feasibility and preliminary effectiveness of an online training and support program for caregivers of people with dementia in India: A randomized controlled trial. Int. J. Geriatr. Psychiatry.

[52] Blackberry I., Rasekaba T., Morgan D., Royals K., Greenhill J., Perkins D., O’Connell M., Hamiduzzaman M., Winbolt M., Robinson A. (2023). Virtual Dementia-Friendly Communities (Verily Connect) Stepped-Wedge Cluster-Randomised Controlled Trial: Improving Dementia Caregiver Wellbeing in Rural Australia. Geriatrics.

[53] Christie H.L., Dam A.E.H., van Boxtel M., Köhler S., Verhey F., de Vugt M.E. (2022). Lessons Learned From an Effectiveness Evaluation of Inlife, a Web-Based Social Support Intervention for Caregivers of People With Dementia: Randomized Controlled Trial. JMIR Aging.

[54] Fossey J., Charlesworth G., Fowler J.-A., Frangou E., Pimm T.J., Dent J., Ryder J., Robinson A., Kahn R., Aarsland D. (2021). Online education and cognitive behavior therapy improve dementia caregivers’ mental health: A randomized trial. J. Am. Med. Dir. Assoc..

[55] Liu Y., Hughes M.C., Baumbach A., Derain L. (2023). An online intervention to improve the health and well-being of informal caregivers of individuals with Alzheimer’s disease: A pilot study. PEC Innov..

[56] Teles S., Ferreira A., Paúl C. (2022). Feasibility of an online training and support program for dementia carers: Results from a mixed-methods pilot randomized controlled trial. BMC Geriatr..

[57] Lorca-Cabrera J., Grau C., Martí-Arques R., Raigal-Aran L., Falcó-Pegueroles A., Albacar-Riobóo N. (2020). Effectiveness of health web-based and mobile app-based interventions designed to improve informal caregiver’s well-being and quality of life: A systematic review. Int. J. Med. Inform..

[58] Amador-Marín B., Guerra-Martín M.D. (2017). Eficacia de las intervenciones no farmacológicas en la calidad de vida de las personas cuidadoras de pacientes con enfermedad de Alzheimer. Gac. Sanit..

[59] Christie H.L., Bartels S.L., Boots L.M.M., Tange H.J., Verhey F.R.J., de Vugt M.E. (2018). A systematic review on the implementation of eHealth interventions for informal caregivers of people with dementia. Internet Interv..

[60] Research Partners (2017). mHealth App Economics 2017/2018. Current Status and Future Trends in Mobile Health.

[61] Gallagher D., Ni Mhaolain A., Crosby L., Ryan D., Lacey L., Coen R.F., Walsh C., Coakley D., Walsh J.B., Cunningham C. (2011). Self-efficacy for managing dementia may protect against burden and depression in Alzheimer’s caregivers. Aging Ment. Health.

[62] Pinquart M., Sörensen S. (2006). Helping caregivers of persons with dementia: Which interventions work and how large are their effects?. Int. Psychogeriatr..

[63] Brodaty H., Arasaratnam C. (2012). Meta-analysis of nonpharmacological interventions for neuropsychiatric symptoms of dementia. Am. J. Psychiatry.

[64] Hofmann S.G., Asnaani A., Vonk I.J., Sawyer A.T., Fang A. (2012). The efficacy of cognitive behavioral therapy: A review of meta-analyses. Cogn. Ther. Res..

[65] Moreno-Cámara S., Palomino-Moral P.Á., Moral-Fernández L., Frías-Osuna A., Parra-Anguita L., del-Pino-Casado R. (2019). Perceived needs of the family caregivers of people with dementia in a mediterranean setting: A qualitative study. Int. J. Environ. Res. Public Health.

[66] Gobierno de Canaria (2012). Manual de Actuación en la Enfermedad de Alzheimer y Otras Demencias.

[67] Aledeh M., Adam H. (2020). Caring for Dementia Caregivers in Times of the COVID-19 Crisis: A Systematic Review. Am. J. Nurs. Res..

[68] Parra-Vidales E., Soto-Pérez F., Perea-Bartolomé M.V., Franco-Martín M.A., Muñoz-Sánchez J.L. (2017). Online interventions for caregivers of people with dementia: A systematic review. Actas Esp. Psiquiatr..

[69] Wasilewski M.B., Stinson J.N., Cameron J.I. (2017). Web-based health interventions for family caregivers of elderly individuals: A Scoping Review. Int. J. Med. Inform..

[70] Andersson G., Titov N. (2014). Advantages and limitations of Internet-based interventions for common mental disorders. World Psychiatry.

